# HydroZitLa inhibits calcium oxalate stone formation in nephrolithic rats and promotes longevity in nematode *Caenorhabditis elegans*

**DOI:** 10.1038/s41598-022-08316-8

**Published:** 2022-03-24

**Authors:** Nalinthip Lordumrongkiat, Nattida Chotechuang, Mani Iyer Prasanth, Depicha Jindatip, Chakriwong Ma-on, Kamonchanok Chuenwisad, Asada Leelahavanichkul, Tewin Tencomnao, Chanchai Boonla

**Affiliations:** 1 Departments of Biochemistry, Faculty of Medicine, Chulalongkorn University, Rama IV Rd, Bangkok, 10330 Thailand; 2 Department of Food Technology, Faculty of Science, Chulalongkorn University, Bangkok, Thailand; 3 Natural Products for Neuroprotection and Anti-Ageing Research Unit, Department of Clinical Chemistry, Faculty of Allied Health Sciences, Chulalongkorn University, Bangkok, Thailand; 4 Departments of Anatomy, Faculty of Medicine, Chulalongkorn University, Bangkok, 10330 Thailand; 5 Department of Pathology, Faculty of Medicine Siriraj Hospital, Mahidol University, Bangkok, Thailand; 6 Departments of Microbiology, Faculty of Medicine, Chulalongkorn University, Bangkok, 10330 Thailand

**Keywords:** Kidney diseases, Risk factors, Drug development, Experimental models of disease, Preclinical research

## Abstract

Low fluid intake, low urinary citrate excretion, and high oxidative stress are main causative factors of calcium oxalate (CaOx) nephrolithiasis. HydroZitLa contains citrate and natural antioxidants and is developed to correct these three factors simultaneously. Antioxidants theoretically can prolong the lifespan of organisms. In this study, we preclinically investigated the antilithogenic, lifespan-extending and anti-aging effects of HydroZitLa in HK-2 cells, male Wistar rats, and *Caenorhabditis elegans*. HydroZitLa significantly inhibited CaOx crystal aggregation in vitro and reduced oxidative stress in HK-2 cells challenged with lithogenic factors. For experimental nephrolithiasis, rats were divided into four groups: ethylene glycol (EG), EG + HydroZitLa, EG + Uralyt-U, and untreated control. CaOx deposits in kidneys of EG + HydroZitLa and EG + Uralyt-U rats were significantly lower than those of EG rats. Intrarenal expression of 4-hydroxynonenal in EG + HydroZitLa rats was significantly lower than that of EG rats. The urinary oxalate levels of EG + HydroZitLa and EG + Uralyt-U rats were significantly lower than those of EG rats. The urinary citrate levels of EG + HydroZitLa and EG + Uralyt-U rats were restored to the level in normal control rats. In *C. elegans*, HydroZitLa supplementation significantly extended the median lifespan of nematodes up to 34% without altering feeding ability. Lipofuscin accumulation in HydroZitLa-supplemented nematodes was significantly lower than that of non-supplemented control. Additionally, HydroZitLa inhibited telomere shortening, p16 upregulation, and premature senescence in HK-2 cells exposed to lithogenic stressors. Conclusions, HydroZitLa inhibited oxidative stress and CaOx formation both in vitro and in vivo. HydroZitLa extended the lifespan and delayed the onset of aging in *C. elegans* and human kidney cells. This preclinical evidence suggests that HydroZitLa is beneficial for inhibiting CaOx stone formation, promoting longevity, and slowing down aging.

## Introduction

Urinary stone disease is an ancient condition in humans documented since the Egyptian era^1,2^. This disease is still a significant urologic problem worldwide with progressively increasing prevalence. Urinary stones are predominantly composed of calcium oxalate (CaOx), mostly formed in the kidneys, and highly recurrent. Although kidney stone is widely perceived as a not life-threatening condition, it has a lethal consequence. Stones progressively damage kidneys and eventually cause chronic kidney disease^3–7^. The economic burden of stone disease is substantial, as the cost of stone treatment and management is comparable with the combined cost of prostate and bladder cancer treatments^8^. Therefore, a new medical prophylaxis for stone formation is needed.

Inadequate fluid intake, decreased urinary citrate excretion, and increased oxidative stress are the three main causative factors of CaOx lithogenesis. These factors should be corrected to prevent CaOx calcification. Fluid intake of 2.5–3 L/day is a general preventive measure^9^. Potassium citrate is a mainstream medication for stone disease because hypocitraturia is the best-known cause of CaOx stone formation and recurrence. Since a long-term treatment with potassium citrate is required to effectively prevent stone disease recurrence, poor adherence becomes an issue because of its side effects and bad/salty taste^10–12^. Taste enhancement by adding sucralose was shown to improve the tolerance of potassium citrate supplementation^13^.

Oxidative stress induced by reactive oxygen species (ROS) is vitally involved in CaOx lithogenesis^14–19^. A low antioxidant diet was demonstrated to contribute to stone formation in experimental rats by increasing ROS production, oxidative stress, and intrarenal CaOx deposition^20^. Antioxidant intervention attenuated oxidative stress and inhibited CaOx deposition in nephrolithic rats^16,21–23^. Medicinal plant-based remedies and traditional medicine for stone treatment fundamentally mitigated oxidative stress^24^.

Traditional herbal medicine or phytomedicine has the advantage of long-standing use in the community, and patients generally consider them as natural and safe regimens. However, it should be noted that scientific evidence of efficacy and safety must be provided prior to clinical implementation. We hypothesized that the regimen combining modern and traditional medicine could be an innovative solution for minimizing side effects without compromising therapeutic efficacy. We developed a new combination of traditional and modern medicine, called HydroZitLa (Hydrozitla or Hyla), as an alternative treatment for CaOx calculi.

The main active antilithogenic ingredients of HydroZitLa are citrate and banana stem water extract. Banana stem (*Musa *spp.) is a long-known Indian Ayurvedic traditional medicine for treating urinary stones^25^, and its stone inhibitory activity has been experimentally demonstrated^26–29^. Poonguzhali and Chegu showed that banana stem extract significantly reduced the urinary oxalate excretion in hyperoxaluric urolithic rats^30^. The preliminary clinical study by Pillai in 1995 showed that the core of banana pseudostem could be used in urolithiasis treatment^31^. Furthermore, banana stem juice has a diuretic effect that could help flush out lithogenic crystals and facilitate stone passage. While potassium citrate corrects only the hypocitraturic risk factor by supplying citrate, HydroZitLa beverage can simultaneously correct all the three aforementioned CaOx risk factors by increasing fluid intake, restoring urinary citrate, and supplying natural antioxidants.

In this study, we investigated whether HydroZitLa could exert the stone inhibitory effect of CaOx in in vitro and in vivo models. The preclinical toxicity of HydroZitLa in human kidney (HK-2) cells and mice was determined. Given that HydroZitLa contained plant-derived antioxidants, we sought to determine whether it could extend the lifespan and reduce the aging phenotype in a *Caenorhabditis elegans* model. We also investigated whether HydroZitLa could inhibit telomere shortening and premature senescence in HK-2 cells.

## Results

### HydroZitLa: a safe beverage for consumption

HydroZitLa was negative for *Clostridium* spp./0.1 g, *Salmonella* spp./25 g, and *Staphylococcus aureus*/0.1 g. Arsenic was not detected. Lead was found at an extremely low level (0.033 ppm). Levels of metals, such as Tl, As, Se, Mo, Zn, Sb, Pb, Cd, Co, Ni, Fe, Mn, Cr, Mg, V, Be, Ca, Cu, Ti, Sr, and Li, in HydroZitLa measured by the inductively coupled plasma-optical emission spectrometry (ICP-OES) are shown in Supplementary Table 1. Toxic metals including Cd, Cr, As, Ni, Tl, Sb, Co, Be, V, and Li were not detected. Trace elements, such as Mg, Zn, Ca, and Fe, were found at 14.04 ± 0.05, 2.09 ± 0.05, 0.86 ± 0.02, and 0.16 ± 0.001 ppm, respectively.

In vitro cytotoxicity of HydroZitLa in HK-2 cells using the 3-(4,5-dimethylthiazol-2yl)-2,5-diphenyltetrazolium bromide (MTT) assay was performed and reported in our previous study^32^. Increased HydroZitLa concentration gradually decreased cell survival. The IC50 of HydroZitLa in HK-2 cells was 24.7% v/v. In vivo acute toxicity testing revealed that all HydroZitLa-administered mice survived until the end of the experiment (14 days). No gross lesions were found in the visceral organs both in the HydroZitLa-administered group and control group. Therefore, the LD50 of HydroZitLa was > 20 mL/kg.

### CaOx aggregation inhibition and antioxidant function of HydroZitLa

**Figure 1:**
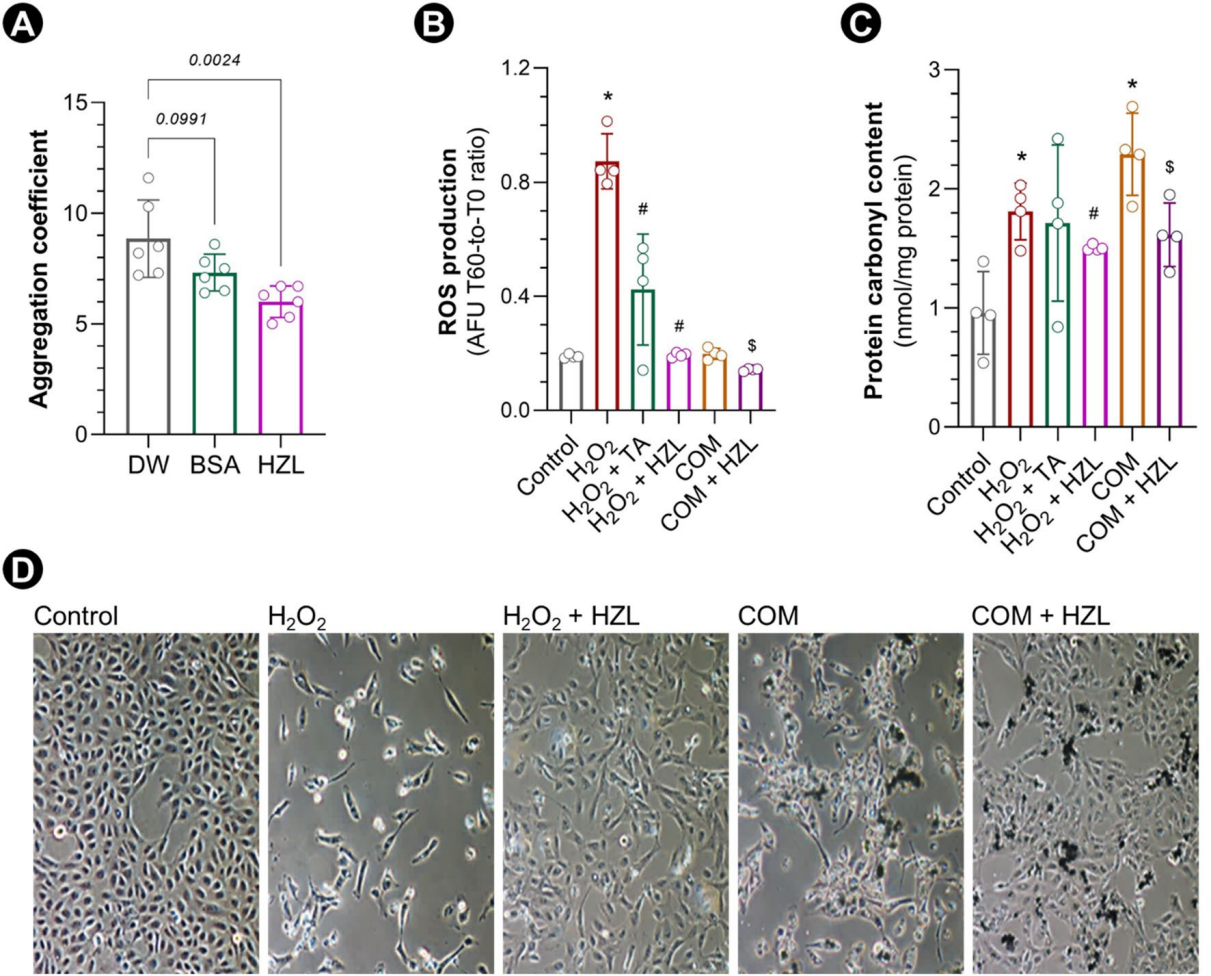
COM aggregation inhibition and oxidative stress mitigation by HydroZitLa. (**A**) HydroZitLa (HZL) significantly decreased aggregation of calcium oxalate monohydrate (COM) crystals compared with distilled water (DW) control. (**B**) H_2_O_2_ (10 µM) significantly increased ROS generation in HK-2 cells compared with control. Co-treatments with tocopheryl acetate (TA, 300 µM) and HydroZitLa (10% v/v) significantly decreased ROS generation in HK-2 cells treated with H_2_O_2_. (**C**) Protein carbonyl contents in HK-2 cells treated with H_2_O_2_ (1,000 µM) and COM (150 µg/cm^2^) were significantly higher than that in control. HydroZitLa co-treatment significantly decreased levels of ROS generation and protein carbonyl content in HK-2 cells treated with H_2_O_2_ and COM. (**D**) Representative micrographs showing reduction of cell survival and induction of morphological change in HK-2 cells treated with H_2_O_2_ (1000 µM) and COM (black precipitates, 150 µg/cm^2^). HydroZitLa co-treatment rescued cells from apoptosis as well as restored cell morphology and proliferation. ^*^P < 0.05 vesrus Control, ^#^*P* < 0.05 versus H_2_O_2_, ^$^*P* < 0.05 versus COM. Micrograph magnification: × 100.

HydroZitLa significantly inhibited the aggregation of seed calcium oxalate monohydrate (COM) crystals, but bovine serum albumin (BSA, 1 mg/mL) did not (Fig. 1A). Citric acid strongly inhibited COM aggregation (Supplementary Fig. 1).

HydroZitLa concentration at 10% (v/v) provided the greatest antioxidative action with the lowest cytotoxicity. Therefore, we used 10% (v/v) HydroZitLa for the subsequent treatment in cell culture model. ROS production in HK-2 cells exposed to H_2_O_2_ and COM was significantly increased compared with control, but it was significantly decreased after HydroZitLa co-treatment (Fig. 1B). In addition, HydroZitLa significantly reduced the levels of protein carbonyl content in HK-2 cells exposed to H_2_O_2_ and COM (Fig. 1C). The exposure of HK-2 cells to H_2_O_2_ (1000 µM) and COM (150 µg/cm^2^) caused changes in cell morphology and increased cell death. HydroZitLa co-treatment prevented morphological change, rescued cells from apoptosis, and restored cell proliferation (Fig. 1D).

### Inhibition of CaOx crystal deposits in rat kidneys

**Figure 2:**
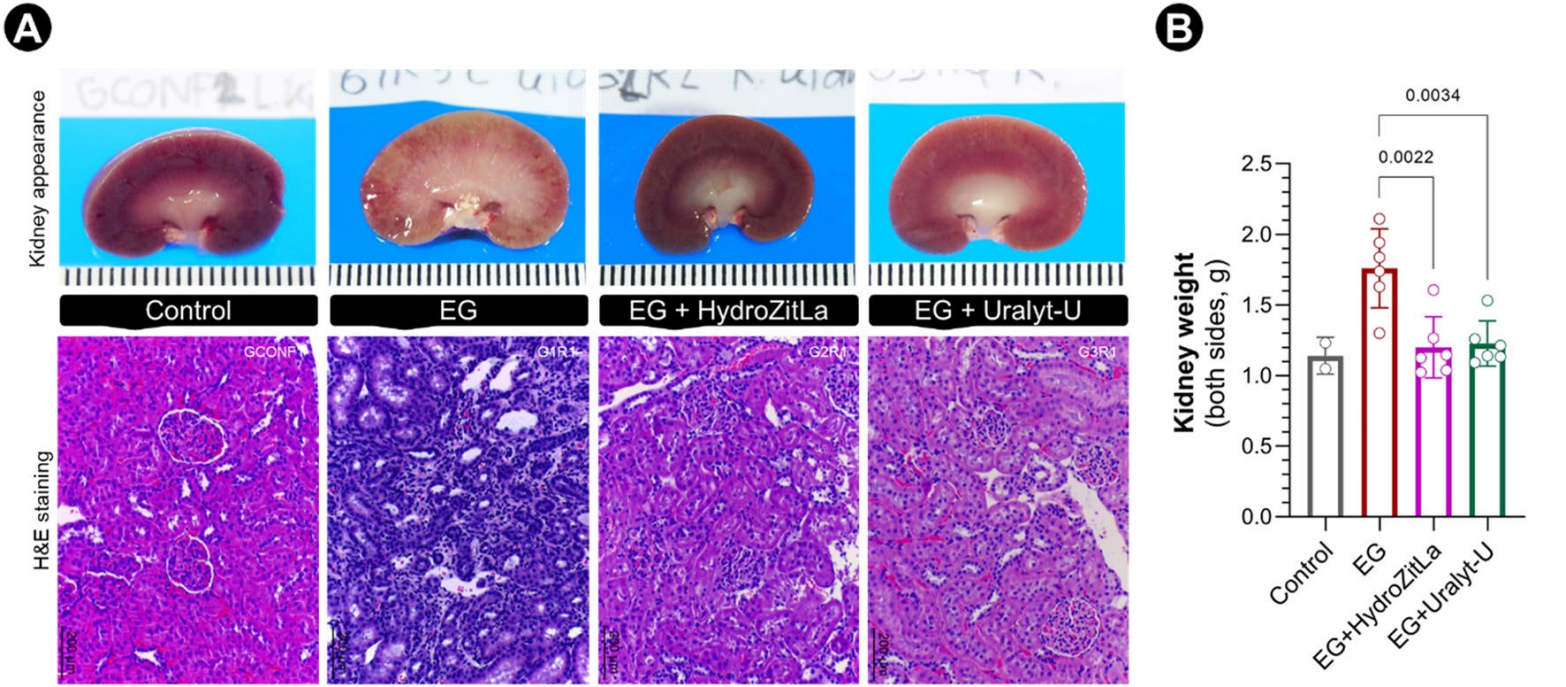
Kidney appearance and H&E staining of rat renal sections. (**A**) Kidney of EG rats were pale, enlarged, and swelled (upper panel). Renal tissues of EG rats had a robust sign of inflammation, but renal tissues of EG + HydroZitLa and EG + Uralyt-U rats appeared normal, similar to that in normal control rats (lower panel). (**B**) The average kidney weights of EG rats were significantly greater than that of EG + HydroZitLa and EG + Uralyt-U rats. Micrograph magnification: × 100.

The biochemical profile of post-intervention 24-h urine samples (day 35) obtained from experimental rats is presented in Table 1. Kidneys of ethylene glycol (EG) rats were enlarged and pale (Fig. 2A), and their weights were significantly greater than that of EG + HydroZitLa and EG + Uralyt-U rats (Fig. 2B). Hematoxylin and eosin (H&E) staining revealed that kidneys of EG rats had a robust sign of inflammation, whereas kidneys of EG + HydroZitLa and EG + Uralyt-U rats appeared normal, similar to that in normal control rats (Fig. 2A). All H&E-stained renal sections are displayed in Supplementary Fig. 2.

**Table 1:** Characteristic of 24-h urine (Day 35) of the experimental rats.

	Control	EG	EG + HydroZitLa	EG + Uralyt-U
Number of rats	2	6	6	6
Body weight at day 0 (g)	223.0 ± 7.7	195.4 ± 21.3	214.1 ± 36.3	203.9 ± 25.4
Body weight at day 35 (g)	371.5 ± 15.8	328.2 ± 30.4	352.8 ± 40.0	349.1 ± 36.1
Urine volume (mL)	23.7 ± 3.2	42.2 ± 18.6	34.1 ± 12.8	39.6 ± 20.9
Urine pH	8.0 ± 0.5	7.7 ± 0.8	7.4 ± 0.6	8.0 ± 0.6
Urine specific gravity	1.030 ± 0	1.170 ± 0.3	1.024 ± 0	1.027 ± 0
Urine creatinine (mg/day)	9.3 ± 5.0	5.1 ± 2.0	7.0 ± 4.1	5.9 ± 5.0
Urine total protein (mg/day)	10.7 ± 1.2	12.4 ± 3.9	13.6 ± 2.6	16.3 ± 3.4

**Figure 3:**
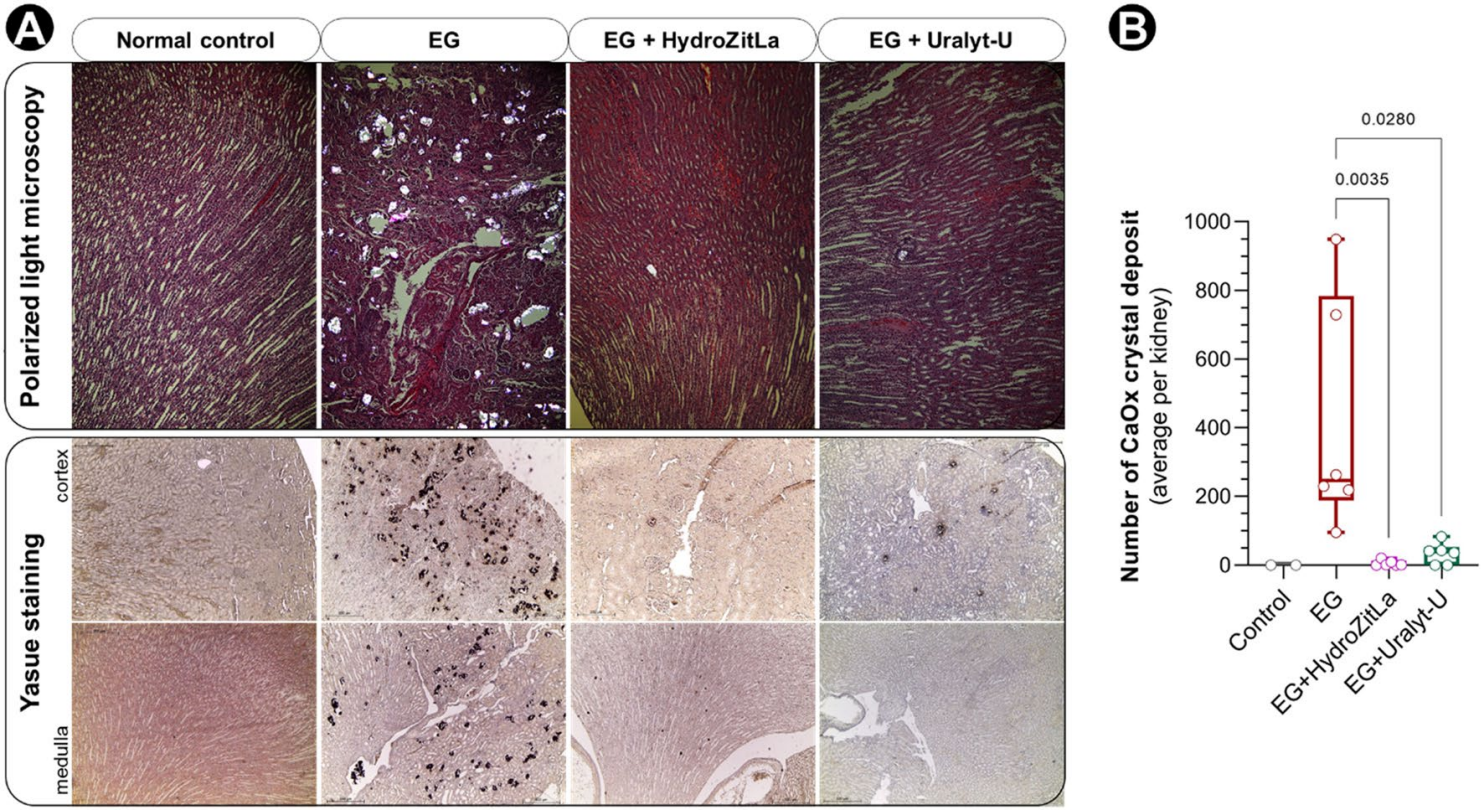
CaOx crystal deposits in rat renal sections detected by polarized light microscopy and Yasue staining. (**A**) The birefringent CaOx crystals were obviously accumulated in the renal sections of EG rats (upper panel). By contrast, the birefringent CaOx deposits were apparently vanished in the kidneys of EG + HydroZitLa and EG + Uralyt-U rats. Yasue staining confirmed that CaOx deposits were substantially deposited in EG rats’ kidneys, but they almost disappeared in kidneys of EG + HydroZitLa and EG + Uralyt-U rats (lower panel). These CaOx deposits were not observed in normal control rats. (**B**) CaOx deposits were counted in Yasue-stained sections. Numbers of intrarenal CaOx deposits in EG + HydroZitLa and EG + Uralyt-U rats were significantly lower than in EG rats. Micrograph magnification: × 100.

Birefringent CaOx crystal deposits were markedly observed in renal sections of EG rats, but they were not found in kidney sections of normal control rats (Fig. 3A). These CaOx deposits almost disappeared in renal sections of EG + HydroZitLa and EG + Uralyt-U rats. Polarized micrographs of all renal sections are shown in Supplementary Fig. 3.

Yasue staining confirmed that CaOx crystals (black precipitates) largely accumulated in the kidneys (in both the cortex and medulla) of EG rats, but this CaOx deposition was remarkably inhibited by HydroZitLa and Uralyt-U (Fig. 3A). The number of black CaOx precipitates in the kidney sections of EG + HydroZitLa and EG + Uralyt-U rats were significantly lower than those in EG rats (Fig. 3B). Results of Yasue staining of all renal sections are shown in Supplementary Fig. 4.

### Reduction of 4-hydroxynonenal (4-HNE) expression in renal tissues

**Figure 4:**
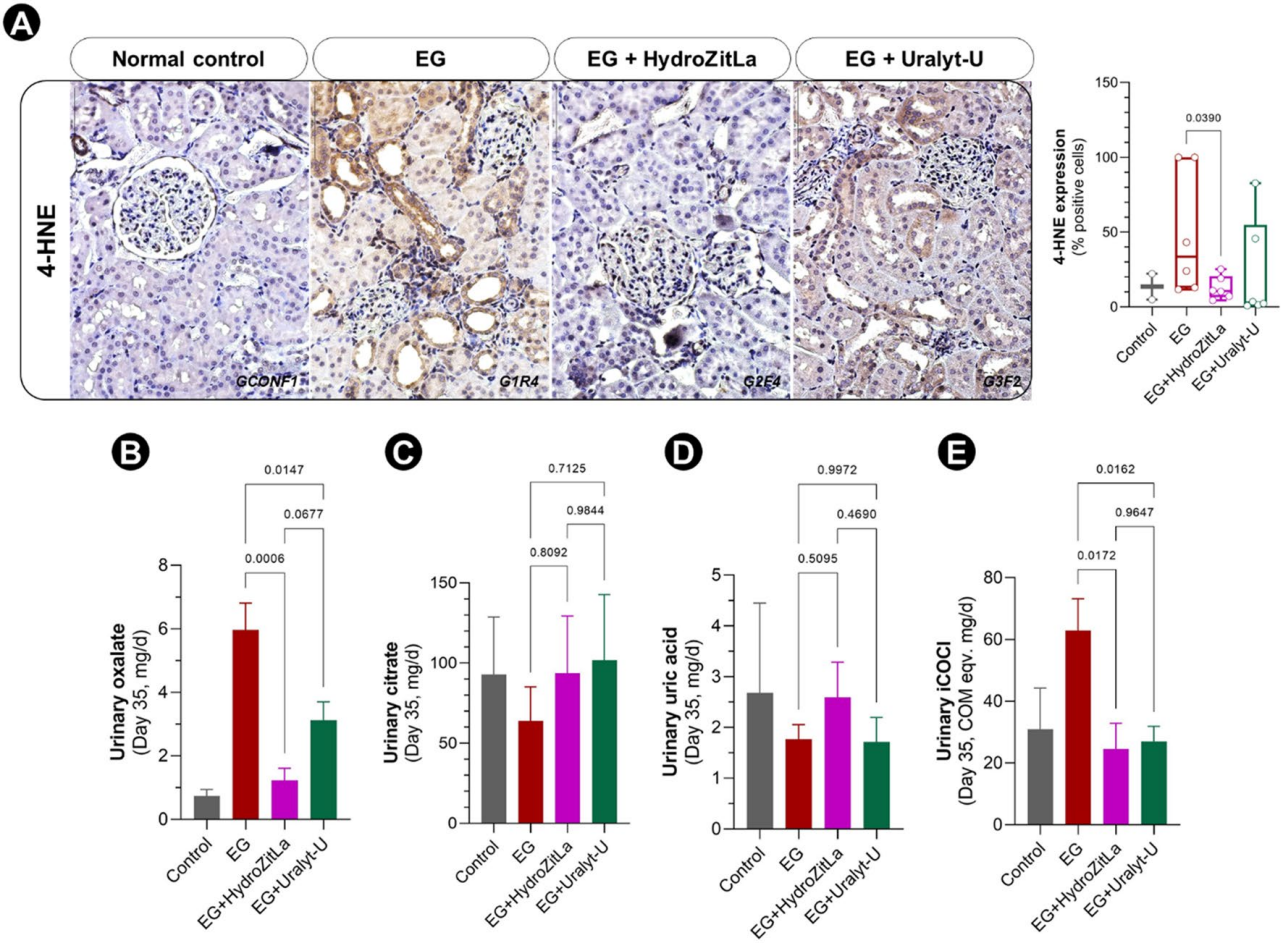
Expression of 4-HNE in rat renal sections and levels of oxalate, citrate, uric acid, and iCOCI in 24-h urine samples (day 35). (**A**) 4-HNE expression was obviously increased in EG rat renal tissues relative to normal control rat. Intrarenal expression of 4-HNE in EG + HydroZitLa rats was significantly lower than that in EG rats. (**B**) Urinary oxalate was elevated in EG rats relative to normal control rats. Levels of urinary oxalate in EG + HydroZitLa and EG + Uralyt-U rats were significantly lower than that in EG rats. (**C**) Urinary citrate was declined in EG rats relative to normal control rats. Levels of urinary citrate excretion in rats treated with HydroZitLa and Uralyt-U were higher than that in EG rats, and they could reach the normal levels found in normal control rats. (**D**) Urinary uric acid levels among EG, EG + HydroZitLa, and EG + Uralyt-U rats were comparable. (**E**) Similar to urinary oxalate, levels of urinary iCOCI of EG + HydroZitLa and EG + Uralyt-U rats were significantly lower than that of EG rats. Micrograph magnification: × 400.

The intrarenal expression of 4-HNE, as an oxidative stress marker, was markedly increased in EG rats compared with normal control rats (Fig. 4A). Overall, the expressions of 4-HNE in the kidneys of EG + HydroZitLa and EG + Uralyt-U rats were lower than that in EG rats. However, quantitatively, the proportion of 4-HNE-positive cells was significantly lower only in EG + HydroZitLa rats than in EG rats (Fig. 4A). Results of 4-HNE staining of all renal sections are presented in Supplementary Fig. 5. In this study, we also performed immnunohistochemical staining for cleaved caspase-3, but we did not observe a significant change of this apoptotic marker among the groups (Supplementary Fig. 6).

### Reduction of urinary oxalate and indole-reacted calcium oxalate crystallization index (iCOCI) levels

Urinary oxalate was elevated in EG rats relative to control rats. This urinary oxalate elevation was significantly reduced after HydroZitLa and Uralyt-U treatments (Fig. 4B). By contrast, urinary citrate was decreased in EG rats relative to control rats. Although HydroZitLa and Uralyt-U treatments did not significantly increase urinary citrate levels relative to EG rats, they could restore urinary citrate levels to the level found in normal control rats (Fig. 4C). Levels of urinary uric acid compared among EG, EG + HydroZitLa, and EG + Uralyt-U groups were not significantly different (Fig. 4D). Similar to urinary oxalate, urinary iCOCI levels of EG + HydroZitLa and EG + Uralyt-U rats were significantly lower than that of EG rats (Fig. 4E).

### *C. elegans* lifespan extension

**Figure 5:**
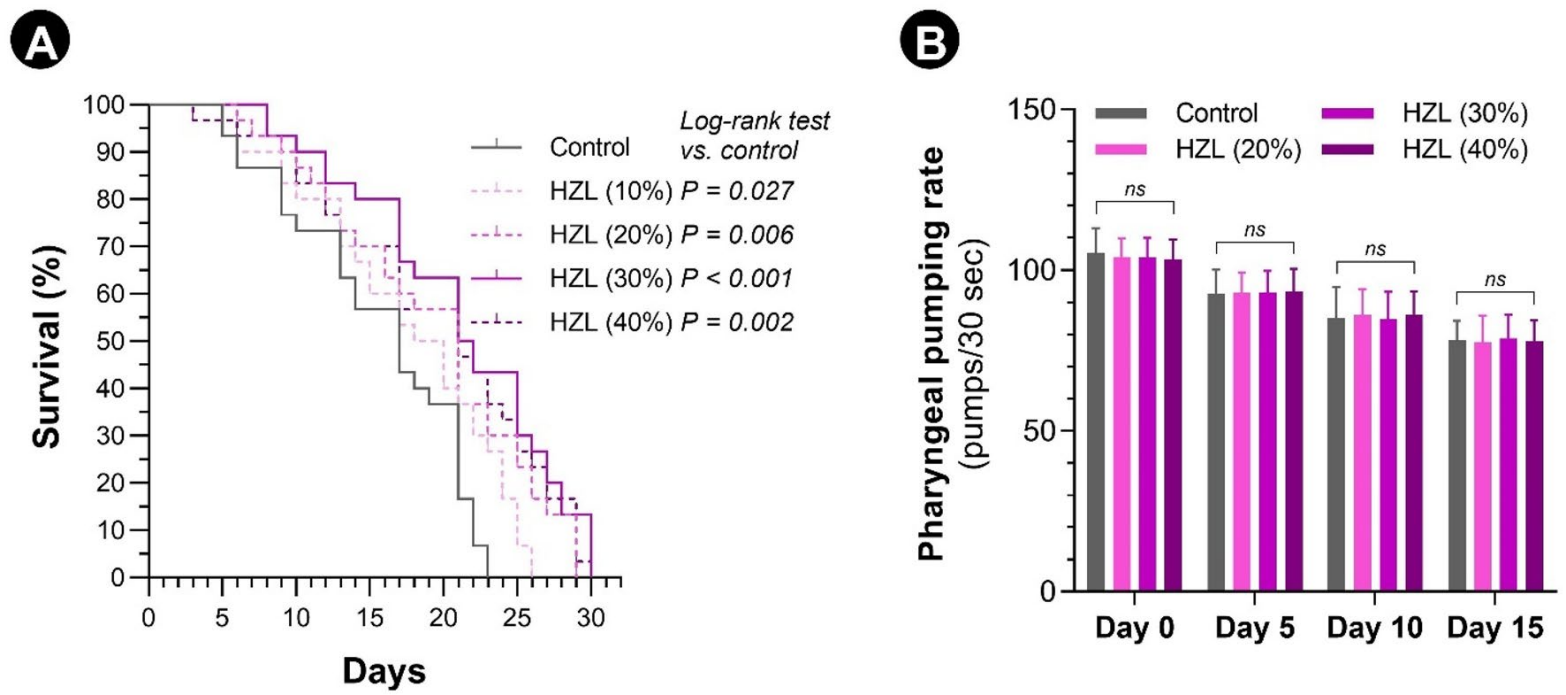
Lifespan extension and food intake behavior of *C. elegans* supplemented with HydroZitLa (HZL). (**A**) Kaplan–Meier survival curves compared between *C. elegans* supplemented with HydroZitLa (10%–40% v/v) and non-supplemented control. Supplementation with 10%, 20%, 30%, and 40% v/v HydroZitLa significantly increased the median lifespan of nematodes compared with non-supplemented control. The highest lifespan extension was with 30% v/v HydroZitLa supplementation. Median survival time of non-supplemented control worms was 16 days. Supplementations with HydroZitLa at 10%, 20%, 30%, and 40% significantly increased the median survivals to 18.5 (15.6% increase), 19.5 (21.9% increase), 21.5 (34.4% increase), and 21.0 (31.3% increase) days, respectively. (**B**) Pharyngeal pumping rates measured at days 0, 5, 10, and 15 were not significantly different between supplemented and non-supplemented worms. NS: not significant.

Supplementation with HydroZitLa (10–40% v/v) significantly extended the maximum lifespan of wild-type *C. elegans* (Fig. 5A). The highest lifespan-extending effect was with 30% v/v, which could increase the median survival of nematodes from 16.0 days (control) to 21.5 days (34.4% increase). The median survival of nematodes supplemented with 10%, 20%, and 40% v/v HydroZitLa were significantly prolonged to 18.5 (15.6% increase), 19.5 (21.9% increase) and 21.0 (32.3% increase) days, respectively. The effect of all tested concentrations of HydroZitLa (from 1 to 100% v/v) on the survival of *C. elegans* is reflected in Supplementary Fig. 7.

Food intake behavior, as indicated by pharyngeal pumping rates, at days 0, 5, 10, and 15 was not significantly different between HydroZitLa-supplemented (20%, 30% and 40% v/v) and non-supplemented control nematodes (Fig. 5B).

**Figure 6:**
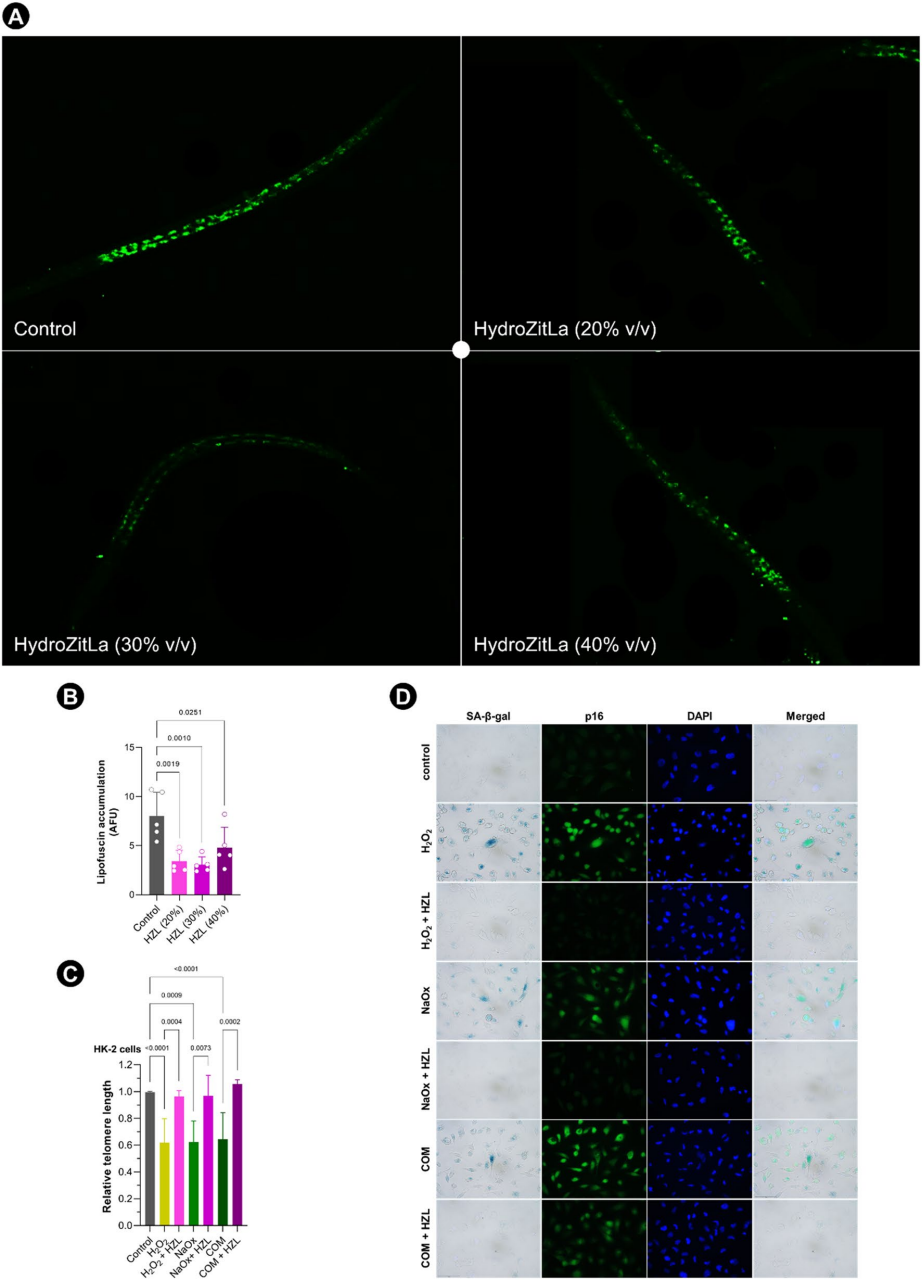
Anti-aging effect of HydroZitLa (HZL) tested in *C. elegans* and HK-2 cells. (**A**) HydroZitLa supplementations at 20%, 30%, and 40% v/v clearly decreased lipofuscin accumulation in *C. elegans*. (**B**) Autofluorescent intensities of lipofuscin in HydroZitLa-supplemented nematodes (for all tested concentrations) were significantly lower than that in non-supplemented control. (**C**) Relative telomere lengths in HK-2 cells treated with H_2_O_2_, sodium oxalate (NaOx), and calcium oxalate monohydrate (COM) were significantly lower than untreated control. HydroZitLa co-treatment significantly inhibited shortening of telomere in HK-2 cells treated with H_2_O_2_, NaOx, and COM. (**D**) Double staining of SA-β-gal and p16 demonstrated that H_2_O_2_, NaOx, and COM induced premature senescence (as indicated by increased proportion of SA-β-gal positive cells) and upregulation of p16 expression. p16 was strongly upregulated in those SA-β-gal positive cells. HydroZitLa co-treatment effectively inhibited upregulation of p16 and onset of premature senescence in HK-2 cells exposed to lithogenic stressors. Magnifications: × 100 (**A**), × 400 (**D**).

The level of autofluorescent lipofuscin (aging marker) was monitored inside the nematodes after supplementations with 20%, 30%, and 40% v/v HydroZitLa (Fig. 6A). HydroZitLa supplementations at all tested concentrations significantly reduced the levels of lipofuscin accumulation in nematodes compared with control (Fig. 6B).

### Inhibition of telomere attrition, p16 upregulation, and premature senescence in HK-2 cells

The relative telomere lengths of HK-2 cells treated with H_2_O_2_, NaOx, and COM were significantly shorter than that of untreated controls (Fig. 6C). HydroZitLa co-treatment significantly impeded the shortening of telomere in HK-2 cells treated with H_2_O_2_, NaOx, and COM.

Double staining results of senescence-associated β-galactosidase (SA-β-gal) and p16 demonstrated that H_2_O_2_, NaOx, and COM induced stress induced premature senescence (SIPS), as indicated by the increased proportion of SA-β-gal-positive cells and p16 upregulation in HK-2 cells (Fig. 6D). p16 was intensively labeled in those SA-β-gal-positive cells. HydroZitLa co-treatment significantly inhibited SIPS (decreased SA-β-gal positivity) and p16 upregulation in HK-2 cells exposed to H_2_O_2_, NaOx, and COM (Fig. 6D).

## Discussion

Progress in the development of drugs for kidney stone disease is comparatively slow, and no new drugs have been clinically implemented for decades^33^. A replacement therapy by alkali citrate is still a widely used medication for prevention of stone recurrence. Gastrointestinal side effects and bad taste are the main drawbacks of potassium citrate treatment that cause poor compliance^11–13^. The EAU urolithiasis guideline stated that the ideal drug should halt stone formation, have no side effects, and be easy to administer, and these elements are important to achieve good compliance^9^. In this study, we developed HydroZitLa to address the drawbacks of potassium citrate and to meet the ideal drug criteria as much as possible. The HydroZitLa beverage has a delicious sour-and-sweet taste. We believed that the poor palatability of potassium citrate could be solved by HydroZitLa similar to reports by Mechlin et al. who demonstrated that the addition of Splenda to potassium citrate helped improve the compliance of potassium citrate therapy^13^.

A low fluid intake is an important risk factor for CaOx stone formation. Patients with a stone disease are recommended to increase their fluid intake to 2.5–3.0 L/day, but it is relatively difficult for them to maintain adherence, especially in the long-term. Current stone medications are made in the form of either powder or tablet. Water intake with these medications is usually inadequate and varied between patients. By contrast, drinking HydroZitLa enabled patients to increase their water intake at least 500 mL with every pouch consumed. With long-term use, HydroZitLa could promote changes in dietary habits toward increasing daily fluid intake. The toxicity assessment data ensured that HydroZitLa was safe for consumption. The HydroZitLa beverage concentrate was officially approved by the Thai Food and Drug Administration (FDA) and locally available in Thailand since 2019. To our knowledge, consumers have not reported toxicity and serious side effects.

HydroZitLa significantly inhibited the aggregation of COM crystals in vitro. Although albumin was reported as an inhibitor of CaOx aggregation^34^, we did not observe the significant inhibition of CaOx aggregation by BSA in our testing system. By contrast, citric acid strongly inhibited COM aggregation in our assay. Plausibly, citric acid contained in HydroZitLa was responsible for the blockage of COM aggregation.

The phenolic content in HydroZitLa was derived from banana stem, blue pea flower, and sappan wood. The antioxidant properties of banana stem^35^, blue pea flowers^36^, and sappan heartwood^37^ extracts are well documented. The present study showed that HydroZitLa significantly reduced the intracellular ROS production and protein oxidation in HK-2 cells exposed to H_2_O_2_ and COM. Furthermore, HydroZitLa profoundly rescued HK-2 cells from apoptotic cell death induced by H_2_O_2_ and COM. These data highlighted that HydroZitLa had an antioxidative function to mitigate oxidative stress in renal tubular cells.

In this study, we demonstrated in a rat model of CaOx nephrolithiasis in which HydroZitLa significantly inhibited CaOx deposition in rats’ kidneys, and its antilithogenic efficacy was equivalent to Uralyt-U drug. Interestingly, HydroZitLa significantly decreased intrarenal 4-HNE expression, but Uralyt-U did not. HydroZitLa contained antioxidants, but Uralyt-U did not. Our data suggested that an alleviation of oxidative stress was an advantage of HydroZitLa over potassium citrate. HydroZitLa and Uralyt-U had comparable efficacy on the reduction of urinary oxalate in nephrolithic rats, although it was more pronounced with HydroZitLa. Significant reduction of urinary iCOCI levels (indicator of the urinary competency of CaOx crystallization^38^) was also observed in rats treated with HydroZitLa and Uralyt-U. These indicated that the potential of CaOx crystallization in the urine was decreased after HydroZitLa and Uralyt-U treatments. HydroZitLa appeared to deliver antilithogenic activity primarily through the reduction of urinary oxalate excretion. The finding was corroborated with the reports by Poonguzhali and Chegu who showed that hyperoxaluric rats treated with aqueous banana stem extract significantly reduced urinary oxalate excretion^30^. Based on the present findings, HydroZitLa induced CaOx stone inhibitory effects equivalent to the currently used potassium citrate, and it could be clinically useful as a new therapeutic alternative for CaOx nephrolithiasis.

A significant increase in urinary citrate levels in nephrolithic rats after HydroZitLa and Uralyt-U treatments was not detected. However, a trend of increased urinary citrate excretion after both treatments was observed. This might be explained by the small sample size (n = 6) or the inadequate dose of citrate (2 mEq per day, equal to 0.4 g/kg per day on average). Yasui et al. used potassium citrate (Uralyt, Nippon Chemiphar, Japan) at 0.5 g/kg (low dose) and 20 g/kg (high dose) per day in their experiments to find a significant increase in urinary citrate levels in experimental rats^39^. Ghaeni et al. treated EG rats with potassium citrate (Sepidaj, Iran) at 2.5 g/kg per day to observe a significant elevation of urinary citrate excretion after treatment^40^. Thus, a low dose of citrate was likely a reason for not finding a significant elevation of urinary citrate in our rat model following HydroZitLa and Uralyt-U treatments. However, HydroZitLa and Uralyt-U treatments could restore the urinary citrate to the levels observed in normal control rats. This urine citrate normalization could contribute, at least in part, to the inhibition of CaOx formation.

Based on our present findings, HydroZitLa exerted the antilithogenic function through at least two pathways. First, it reduced urinary oxalate excretion, decreased urinary CaOx crystallization capacity, and supplied citrate. Second, it contained herbal polyphenols that were capable of attenuating oxidative stress and inflammation, hence inhibiting the CaOx lithogenesis. Fundamentally, oxalate is endogenously synthesized in the liver using glyoxylate as a prominent precursor^41^. Glyoxylate is synthesized from glycolate by the glycolate oxidase (GO) enzyme, and it is further converted to oxalate by lactate dehydrogenase (LDH). We found that HydroZitLa significantly reduced urinary oxalate excretion in EG-treated rats. We speculated that HydroZitLa inhibited oxalate synthetic pathway in the liver through downregulation of hepatic enzymes involved in the glyoxylate metabolism, particularly LDH and GO. The previous study by Kailash and Varalakshmi demonstrated that banana stem extract reduced the hepatic GO and LDH levels in sodium glycolate-induced hyperoxaluric rats^27^. Poonguzhali and Chegu also reported that crude extract of banana stem reduced urinary oxalate, glycolic acid, and glyoxylic acid in the glycolate-induced hyperoxaluric rats^30^. Recently, Patankar et al. demonstrated that their herbal formulation, called Herbmed Plus (containing banana stem), reduced the level of LDH activity in the liver and kidney tissues^42^. To verify our speculation, expression of hepatic and kidney enzymes involved in oxalate synthesis in EG rats treated with HydroZitLa should be further explored.

Several lines of evidence strongly support that oxidative stress, inflammation, and renal tubular injury are critically involved in the CaOx lithogenesis^3,4,15,43–45^. Oxidative stress induced by oxalate and/or CaOx crystals causes renal tubular injury that creates the sites for crystal nucleation, adhesion, retention, aggregation and finally a nidus formation^43,46,47^. Furthermore, oxidative stress activates the inflammatory reaction that in turn accelerates the lithogenic process^4,47,48^. Thereby, phytomedicine that has antioxidant and anti-inflammatory properties is a promising measure for prevention of kidney stone formation^24^. Aggarwal et al. demonstrated in the EG-induced nephrolithic rat model that bergenin (bioactive phenolic compound isolated from *Bergenia ligulata*) had a potent antilithogenic effect to mitigate oxidative stress, ameliorate mitochondrial dysfunction and decrease tubular injury and inflammation^49^. Rutin and curcumin (the well-known indigenous phytochemicals with antioxidant and anti-inflammatory properties) were shown to inhibit tubulointerstitial damage and crystal deposits in the EG-induced nephrolithic rats^50^. Polyphenols from grape seeds was also demonstrated to inhibit intrarenal CaOx crystal deposits in the EG-induced lithiasis rats, plausibly through their antioxidant activity^51^. An Ayurveda herbal formulation Herbmed was demonstrated to reduce inflammatory injury and crystal deposits in the kidneys of EG-induced nephrolithic rats^42^. In this study, we showed that HydroZitLa decreased intrarenal oxidative stress, inflammation and CaOx crystal deposits, suggested that HydroZitLa exerted antioxidant and anti-inflammatory actions to prevent CaOx renal stone formation.

Several plant polyphenols have been experimentally proven to be beneficial for extending lifespan and delaying aging^52^. Resveratrol is the most well-characterized lifespan-extending polyphenolic compound. It primarily acts through sirtuin activation and oxidative stress attenuation^53^. The longevity effect of resveratrol tested in *C. elegans* model varies among studies with an increase in median lifespan ranging from 10 to 65%^54,55^. HydroZitLa significantly extended the lifespan of wild-type *C. elegans* up to 34% with no sign of toxicity, suggesting that the lifespan-extending effect of HydroZitLa was comparable with that of resveratrol. Dietary restriction is a known key mechanism in longevity^56^. The pharyngeal pumping rates compared between HydroZitLa worms and control worms were comparable, indicating that the lifespan extension by HydroZitLa was not a result of dietary restriction.

HydroZitLa significantly decreased an accumulation of the autofluorescent age pigment lipofuscin in *C. elegans*, suggesting an anti-aging effect. ROS and oxidative stress are well-known inducers of SIPS, and oxidative stress accelerates the shortening of telomeres^57^. We recently demonstrated that oxalate, COM, and urine from patients with CaOx stones induced premature senescence, p16 upregulation and telomere attrition in renal tubular cells through oxidative stress^58^. In this study, we showed that HydroZitLa inhibited telomere attrition, p16 upregulation, and premature senescence in HK-2 cells exposed to H_2_O_2_, oxalate, and COM. Although the molecular mechanism for this anti-aging effect was not elaborated, HydroZitLa was suggested to mechanistically prevent premature senescence through inhibition of telomere shortening and downregulation of p16 expression.

Although herbal remedies and phytomedicines progressively become popular and globally accepted, scientific evidence of efficacy and safety must be provided and should not be compromised. Basically, the herb-drug interaction is hardly predicted because it is greatly varied among individuals, depending on several factors such as polymorphisms of cytochrome P450 enzyme and drug transporters, and existing medical conditions^59^. Adverse effects of herb and medicine coadministration must be closely monitored to identify the potential herb-drug interactions. The best-known herbs seriously interacted with drugs include garlic, Baizhi, Danggui, ginseng, ginkgo, licorice, St John’s wort, Kava, black and long pepper, Danshen, Huangqin and milk thistle^59^. To our knowledge, there have been no serious side effects or drug interactions reported for banana stem, butterfly blue pea flower and sappan wood. In Thailand, banana stem is consumed as food ingredient. Drinking too much (over-consumption) banana stem juice may cause allergy, abdominal pain, and vomiting, thus moderate consumption is recommended. The blue pea flower is commonly used in food as natural colorant, and it is also used for making tea. Adverse effect of over-consumption of butterfly blue pea flower tea, for instances, upset stomach, nausea, and allergic reactions, is rarely reported. Sappan wood is also frequently used as natural food colorant, and in communities (including communities in Thailand) it is commonly consumed as healthy drink. However, the contraceptive function (anti-fertility in males) of sappan wood extract is demonstrated in rat model, as it interferes the spermatogenesis^60^. On the other side, the heartwood of *Caesalpinia sappan L.* is used as a medicinal treatment for gynecological symptoms, including algomenorrhea and amenorrhea^61^. Therefore, safety of the long-term consumption of HydroZitLa in human remains to be ascertained in the clinical trial.

This study has some limitations. The precise active compounds responsible for CaOx stone inhibitory function were not identified and characterized. The exact mechanisms of HydroZitLa to inhibit CaOx stone formation in rats was not extensively investigated. The dose-dependent antilithogenic effect of HydroZitLa was not investigated. The dose of HydroZitLa that could significantly increase urinary citrate level in the nephrolithic rats was not explored. The phenolic profile of HydroZitLa should be further investigated. The mechanism of longevity promotion by HydroZitLa in *C. elegans* was not intensively explored. Finally, specific compounds in HydroZitLa responsible for longevity and anti-aging were not identified and characterized.

In conclusion, HydroZitLa was developed to simultaneously normalize risk factors of CaOx stone formation by supplying fluid, citrate, and natural antioxidants. Experimentally, HydroZitLa inhibited intrarenal CaOx deposit in nephrolithic rats. The CaOx stone inhibitory efficacy of HydroZitLa was equivalent to the currently used potassium citrate (Uralyt-U). HydroZitLa efficiently attenuated oxidative stress both in vitro and in vivo. Remarkably, HydroZitLa extended the lifespan and delayed the onset of aging in *C. elegans*. Furthermore, HydroZitLa significantly inhibited telomere shortening, p16 expression, and premature senescence in human kidney cells. These preclinical findings suggested that HydroZitLa was a clinically promising alternative for CaOx nephrolithiasis treatment accompanied by longevity promotion and anti-aging benefits. Clinical trials are necessary to warrant the therapeutic efficacy of HydroZitLa in humans.

## Materials and methods

### HydroZitLa production, antioxidant content, and nutrition fact

HydroZitLa is a medicinal plant-derived beverage concentrate locally available in Thailand^32^. The beverage is prepared by diluting HydroZitLa concentrate (55 mL in a sealed pouch) with drinking water (to the final volume of 500 mL). It has pinkish purple color with a delicious sour-and-sweet taste. The main ingredients of HydroZitLa were banana stem (*Musa sapientum L.*) water extract, and citric acid (16 mEq/pouch). Traditional Thai medicinal plants including *Clitoria ternatea L.* (Butterfly blue pea flower powder) and *Caesalpinia sappan L.* (Sappan wood powder) were used as natural colorants. Stevia and sucralose were sweeteners. The product was approved by the Thai FDA on April 10, 2019, in the category of beverage in a sealed container (Food serial no. 10–1-02,244–5-0005).

The total antioxidant capacity (TAC) of HydroZitLa was 21.87 ± 7.96 mg vitamin C equivalent antioxidant capacity per pouch (Supplementary Fig. 8). The total phenolic content (TPC) was 19.99 ± 0.83 mg gallic acid equivalent/100 g. The total flavonoid content (TFC) was 4.59 ± 0.31 mg catechin equivalent/100 g. For quality control, TAC, TPC and TFC were used as quality control parameters. The levels of TAC, TPC and TFC in banana stem, butterfly blue pea flower and sappan wood extracts between lots and storage time points were not significantly different. In the production of HydroZitLa, the TAC, TPC, TFC and brix value (17 ± 2) were used as quality control parameters to maintain the consistency between batches.

The total energy of HydroZitLa per pouch (serving) was 25 kcal. No protein, fat, and cholesterol contents were detected. The total carbohydrate was 6 g, sugar was < 1 g, and sodium was 10 mg. The potassium content was 240 mg/100 g. Soluble and insoluble dietary fibers were 0.74 and 0.43 g/100 g, respectively. The pH of HydroZitLa was 3.4.

### Toxicity assessment

The presence of *Escherichia coli*, *Clostridium* spp., *Salmonella* spp., and *Staphylococcus aureus* in HydroZitLa were analyzed by the Bureau of Quality and Safety of Food, Ministry of Public Health, Thailand. The metal profile of HydroZitLa was analyzed by ICP-OES.

The in vivo acute toxicity of HydroZitLa in mice was tested by the Medicinal Plant Research Institute, Ministry of Public Health, Thailand. HydroZitLa or water (control) was orally administered to mice at a dose of 20 mL/kg (n = 10 per group). Mice were observed for 14 days before necropsy. Gross lesions in visceral organs were examined and compared between the HydroZitLa and control groups.

### CaOx aggregation assay

Seed COM crystals were prepared^62^ and diluted to 50 mg/mL in 0.05 M Tris/0.15 NaCl, pH 6.5. HydroZitLa, or BSA, or distilled water (blank) (200 µL) was added to the freshly prepared working seed COM suspension (2 mL). After mixing, absorbance at 620 nm was measured as baseline (AT_0_). After incubation at 37 °C for 10 min, the absorbance at 620 nm was measured again (AT_10_). The aggregation coefficient (AC) was calculated as follows: AC = ((AT_0_ − AT_10_)/10) × 1000. A higher AC value indicates a higher COM aggregation.

### Cell line, cytotoxicity, and intracellular ROS determination

HK-2 cells (ATCC, VA, USA) were maintained in DMEM supplemented with 10% fetal bovine serum and 1% Pen-Strep under 37 °C, 5% CO_2_, and 95% humidity. The cytotoxicity of HydroZitLa in HK-2 cells was assessed by the MTT assay^63^. HK-2 cells were seeded (200 cells/well), grown overnight in 96-well plate, and treated with varied concentrations of HydroZitLa (0 as control, 1.25%, 2.5%, 5%, 10%, 20%, 40%, 50%, 80%, and 100% v/v) for 24 h. After washing, the MTT solution was added, incubated for 1 h, and then discarded. Dimethyl sulfoxide was added to solubilize formazan crystals. Absorption at 570 nm was measured. Cell viability (%) was calculated using untreated cells as control (100% viability).

The inhibition of intracellular ROS production by HydroZitLa was determined by dichloro-dihydro-fluorescein diacetate (DCFH-DA) assay in a 96-well plate^64,65^. HK-2 cells were combined with 0.1 M DCFH-DA solution and incubated at 37 °C for 30 min. After washing, cells were challenged with H_2_O_2_ or COM (with and without 10% v/v HydroZitLa). Fluorescent intensity (excited at 485 nm and emitted at 535 nm) was measured at the beginning (T_0_) and at 60 min (T_60_). Arbitrary fluorescent unit (AFU), which indicated the level of ROS generation, was calculated as follows: AFU = T_60_/T_0_.

### Protein carbonylation measurement

Protein carbonyl content, as an indicator of protein oxidation, was measured according to our earlier reports^64–66^. The treated HK-2 cells (in 100-mm dish) were lysed by radioimmunoprecipitation assay buffer containing protease inhibitor cocktail. Total protein concentration was measured by Bradford assay. The cell lysate was incubated with 10 mM dinitrophenylhydrazine (DNPH) solution or 2 N HCl for 1 h in the dark. Cold 20% trichloroacetic acid was added and incubated for 10 min on ice. Pellets were collected by centrifugation, washed with ethyl acetate:ethanol (1:1), and re-dissolved with 6 M guanidine HCl. Absorbance (A) at 375 nm was measured. The protein carbonyl content normalized by the total protein concentration was calculated as follows: ((A_DNPH_ – A_HCl_) × 45.45)/protein concentration.

### Rat model for CaOx nephrolithiasis

EG (1% v/v) supplemented in drinking water (free access ad libitum, for 35 days) was used for the induction of CaOx nephrolithiasis in rats. Male Wistar rats (6–8 weeks old, 180–300 g) were divided into four groups, namely, EG (n = 6), EG + HydroZitLa (n = 6), EG + Uralyt-U (n = 6), and normal control (n = 2) groups. All rats were housed in stainless-steel cages at 25 °C under 12:12 h light–dark cycle. Two control rats were purposely used only for the comparison of CaOx deposit in rat kidneys and immunohistochemistry, not for urine chemistry. HydroZitLa and Uralyt-U were force-fed twice daily (morning and evening) at a total citrate dose of 2 mEq per day. This administered dose of citrate was equivalent to 60.8 mEq citrate in human calculated based on the dose conversion proposed by Reagan-Shaw et al.^67^. 24-h urine specimens were collected at the end of intervention (day 35) using thymol as a preservative. Rats were anesthetized with isoflurane and sacrificed. Both kidneys were removed, cut, and fixed in 10% neutral formalin buffer for histology study.

All rats were purchased from the National Laboratory Animal Center, Salaya Campus Mahidol University, Nakhonpathom, Thailand. The experimental procedures were conducted in accordance with the guidelines for experimental animals by National Research Council of Thailand and approved by the Institutional Animal Care & Use Committee, Faculty of Medicine, Chulalongkorn University (No. 022/2560). Our study was performed in compliance with the ARRIVE guidelines.

### CaOx deposition in renal sections by polarized microscopy

Formalin-fixed paraffin-embedded rat kidney sections were prepared by automated tissue processor. H&E staining was performed according to the standard procedure. Birefringent CaOx crystal deposits in H&E-stained sections were visualized using a polarized light microscope (OLYMPUS BX50, Japan).

### Yasue staining for CaOx histochemistry

Renal tissue sections were deparaffinized, rehydrated, submerged in 5% acetic acid for 30 min to remove calcium phosphate and calcium carbonate, and washed with distilled water^68^. Sections were incubated with 5% AgNO_3_ for 12 min, washed, and incubated with saturated rubeanic acid in 10% ammonium for 1 min. The stained sections were rinsed with 50% ethanol, washed with distilled water, dehydrated, cleared, and mounted. Black CaOx precipitates were visualized under a light microscope^69^.

### Immunohistochemical staining

After deparaffinization and rehydration, antigen retrieval was performed by boiling in sodium citrate buffer using a microwave. Endogenous H_2_O_2_ was inactivated by incubating with 0.3% H_2_O_2_ for 30 min. Nonspecific binding was blocked by incubating with normal horse serum for 20 min. Sections were then incubated with 1:1,000 4-HNE (ab46545, Abcam, Cambridge, UK) or cleaved caspase-3 (5A1E, Cell Signaling Technology, MA, USA) primary antibody at 4 °C overnight, followed by incubation with secondary antibody at 37 °C for 30 min. After washing, sections were incubated with ABC reagent (VECTASTAIN® ABC kit) for 30 min, soaked in 1% diaminobenzidine staining reagent for 5 min, and counterstained with hematoxylin for 5 min. Finally, sections were dehydrated, cleared, mounted, and visualized under a light microscope.

### Measurements of urinary citrate, oxalate, uric acid, and iCOCI

The urinary level of citrate was determined by high-performance liquid chromatography (Varian Medical Systems, CA, USA) using ROA-organic Acid H ^+^column (300 × 7.8 mm) (Phenomenex, CA, USA), and eluted by 5 mM H_2_SO_4_ at a flow rate of 0.5 mL/min. Urinary oxalate and uric acid levels were measured by capillary electrophoresis (Beckman Coulter, CA, USA) separated at 25 °C with voltage of − 20 kV. Urinary iCOCI test was performed according to our previous report^38^.

### C. elegans strain and maintenance

The *C. elegans* wild-type strain Bristol N_2_ and its laboratory food source *E. coli* OP50 were obtained from the Caenorhabditis Genetics Center, University of Minnesota, USA. They were grown and maintained in nematode growth medium agar plates with a lawn of *E. coli* OP50 at 20 °C^70^. All experiments were conducted in age-synchronized young adult worms.

### Lifespan assay

Lifespan analysis was performed in liquid media^71^. Briefly, 10 age-synchronized young adult nematodes were transferred into a 24-well plate with M9 buffer along with *E. coli* OP50 and 5-fluoro-2′-deoxyuridine for prevention of progeny production. Various concentrations of HydroZitLa were added using distilled water as vehicle control. Alive nematodes were counted every 24 h. Nematodes were considered dead when they did not respond to gentle prodding using the platinum loop.

### Pharyngeal pumping assay

Pharyngeal pumping (indicator of food intake ability) was measured in young adult nematodes (~ 10) compared between control and HydroZitLa supplementation, by monitoring the pharyngeal contraction for 30 s in every 24 h under the stereomicroscope (SMZ-171, Motic, China) on days 0 (before supplementation), 5, 10, and 15^72^.

### Lipofuscin assay

The accumulation of lipofuscin in the wild-type nematodes after supplementation with various concentrations of HydroZitLa (20%, 30%, 40% v/v) for 5 days was monitored. *E. coli* OP50-fed worms were used as control. After the intervention, worms were washed using M9 buffer several times and placed on a glass slide into a drop of sodium azide. Fluorescent imaging was performed using ZEISS LSM 700 confocal microscope. Images were further analyzed using Image J software, and the fluorescence intensity was presented as arbitrary units (AU)^72^.

### Measurement of relative telomere length

The relative telomere length (RTL) was measured in HK-2 cells treated with H_2_O_2_ (25 µM), sodium oxalate (NaOx, 900 µM), and COM (25 µg/cm^2^) with or without HydroZitLa (10% v/v). The RTL was determined by real-time quantitative polymerase chain reaction (qPCR)^73^. It is calculated based on the ratio of the copy number of telomeric repeats to the copy number of a single-copy gene (*36B4* gene) that is proportional to the average telomere length. The primers used were as follows: Telomere (forward) 5′-CGGTTTGTTTGGGTTTGGGTTTGGGTTTGGGTTTGGGTT-3′, Telomere (reverse) 5′-GGCTTGCCTTACCCTTACCCTTACCCTTACCCTTACCCT-3′, 34B4 (forward) 5′-CAGCAAGTGGGAAGGTGTAATCC-3′, and 36B4 (reverse) 5′-CCCATTCTATCATCAACGGGTACAA-3′. PCR was amplified at 95 °C for 10 min, followed by 40 cycles of 95 °C for 15 s and 54 °C for 1 min.

### Double staining of SA-β-gal and p16

HK-2 cells grown on coverslips were treated with H_2_O_2_, NaOx, and COM with and without 10% v/v HydroZitLa for 72 h for induction of SIPS. After treatment, cells were fixed and stained with the freshly prepared X-gal solution (Vivantis, Malaysia) for 12–16 h, washed, permeabilized with 0.1% Triton X-100 (Amresco, TX, USA) for 3 min, and blocked for nonspecific binding with 1% normal horse serum (Gibco, MA, USA) at 37 °C for 1 h. Cells were then incubated with 1:10,000 p16 primary antibody (ab108349, Abcam) at 4 °C overnight, followed by 1:10,000 Alexa Fluor® 488-conjugated secondary antibody (Cell Signaling Technology) at 37 °C for 30 min. The stained coverslip was mounted with Fluoroshield mounting medium with 4′,6-diamidino-2-phenylindole (Abcam). SA-β-gal-positive senescent cells (blue) and p16-expressing cells (green) were visualized and imaged using the EVOS FL Auto 2 imaging system (Thermo Fisher Scientific, MA, USA).

### Statistical analysis

All data were presented as mean ± standard deviation or median (interquartile range), as appropriate. Two-sample *t* test or Mann–Whitney test was performed to test the difference between the two groups. One-way analysis of variance or Kruskal–Wallis test, followed by multiple comparison test, was used to test the difference among three or more groups. GraphPad Prism Software version 9 (GraphPad Software Inc., CA, USA) was employed for computation and graphs. *P* < 0.05 was considered significant.

## Supplementary Information

Supplementary Information.

## Abbreviations

BSA: Bovine serum albumin
CaOx: Calcium oxalate
COM: Calcium oxalate monohydrate
EG: Ethylene glycol
H_2_O_2_: Hydrogen peroxide
HZL: HydroZitLa
iCOCI: Indole-reacted calcium oxalate crystallization index
NaOx: Sodium oxalate
ROS: Reactive oxygen species
RTL: Relative telomere length
SIPS: Stress-induced premature senescence
TA: Tocopheryl acetate
TAC: Total antioxidant capacity
4-HNE: 4-Hydroxynonenal
