# Supplementary material for: HydroZitLa inhibits calcium oxalate stone formation in nephrolithic rats and promotes longevity in nematode Caenorhabditis elegans
Source: Sci Rep. 2022 Mar 24;12:5102. doi: 10.1038/s41598-022-08316-8 (PMC8948263; doi:10.1038/s41598-022-08316-8)

**Supplementary Information**

**Supplementary Figures and Tables**

**Supplementary Table 1** Levels of metals in HydroZitLa measured by inductively couple plasma-optical emission spectrometer (ICP-OES).

| **Metals** | **Concentration (ppm, mg/L)** |
| --- | --- |
| Thallium (Tl) | 0 |
| Arsenic (AS) | UD |
| Selenium (Se) | 0.0043 ± 0.0103 |
| Molybdenum (Mo) | 0.0113 ± 0.0050 |
| Zinc (Zn) | 2.0897 ± 0.0523 |
| Antimony (Sb) | UD |
| Lead (Pb) | 0.0004 ± 0.0006 |
| Cadmium (Cd) | 0 |
| Cobalt (Co) | UD |
| Nickel (Ni) | UD |
| Iron (Fe) | 0.1587 ± 0.0011 |
| Manganese (Mn) | 0.0803 ± 0.0010 |
| Chromium (Cr) | UD |
| Magnesium (Mg) | 14.0420 ± 0.0511 |
| Vanadium (V) | UD |
| Beryllium (Be) | 0 |
| Calcium (Ca) | 0.8627 ± 0.0240 |
| Copper (Cu) | UD |
| Titanium (Ti) | 0.0353 ± 0.0004 |
| Strontium (Sr) | 0.0080 ± 0.0001 |
| Lithium (Li) | 0 |
| *UD: undetectable, Data presented as mean ± SD.* | |

**Supplementary Figure 1** Inhibitory activity of various concentrations of citric acid on COM aggregation. Citric acid at concentrations of 0.5 mM and thereafter higher concentrations significantly inhibited aggregation of COM crystals.


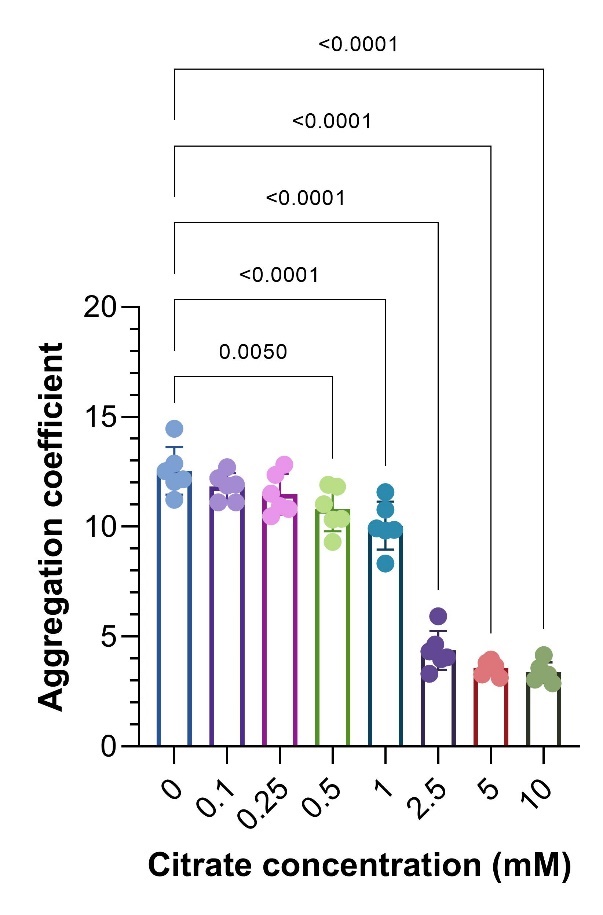


**Supplementary Figure 2** Representative H&E micrographs of all four groups of rat renal sections. Magnification: x100.


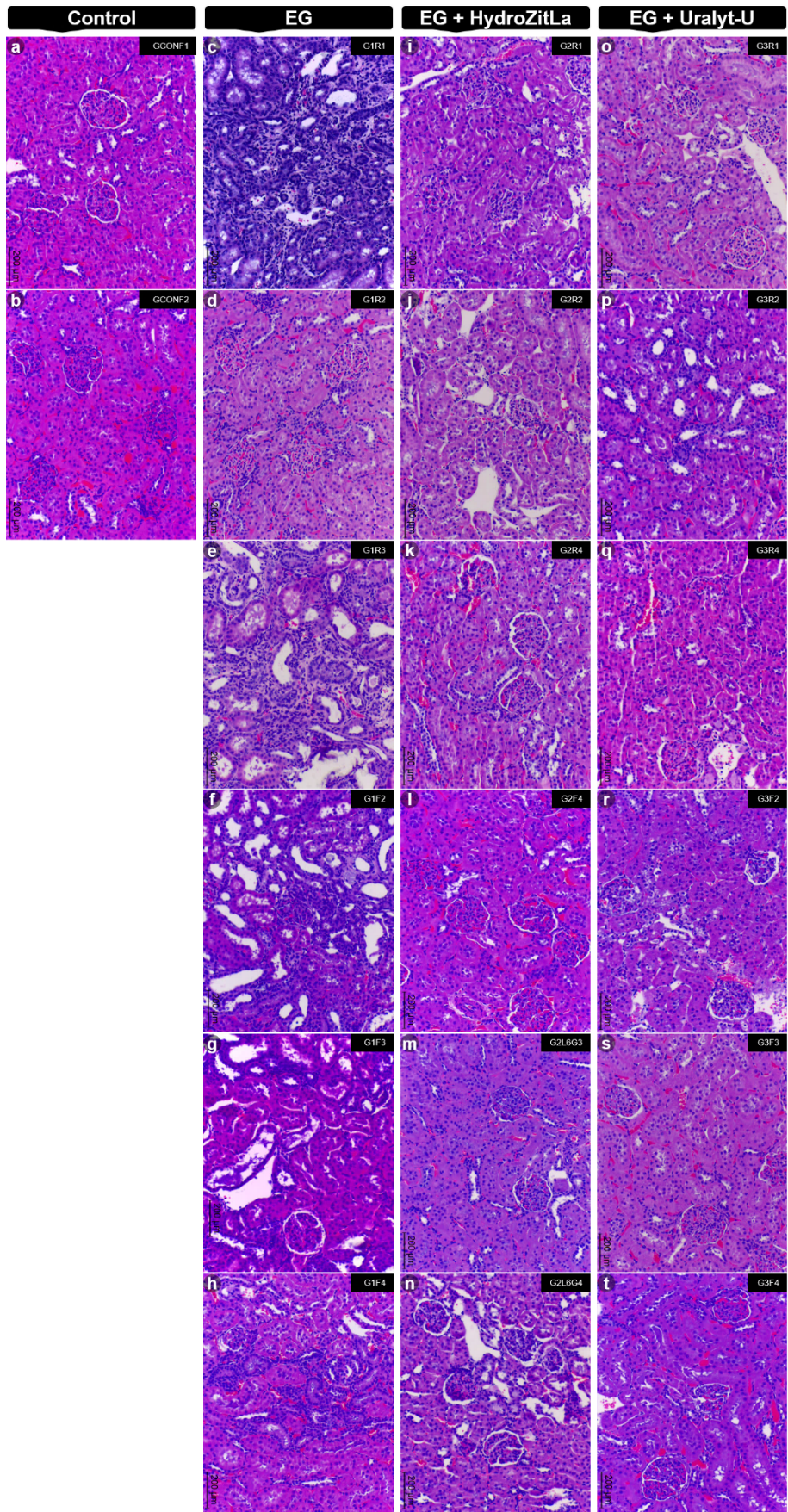


**Supplementary Figure 3** Polarized micrographs of all four groups of rat renal sections. Magnification: x100.


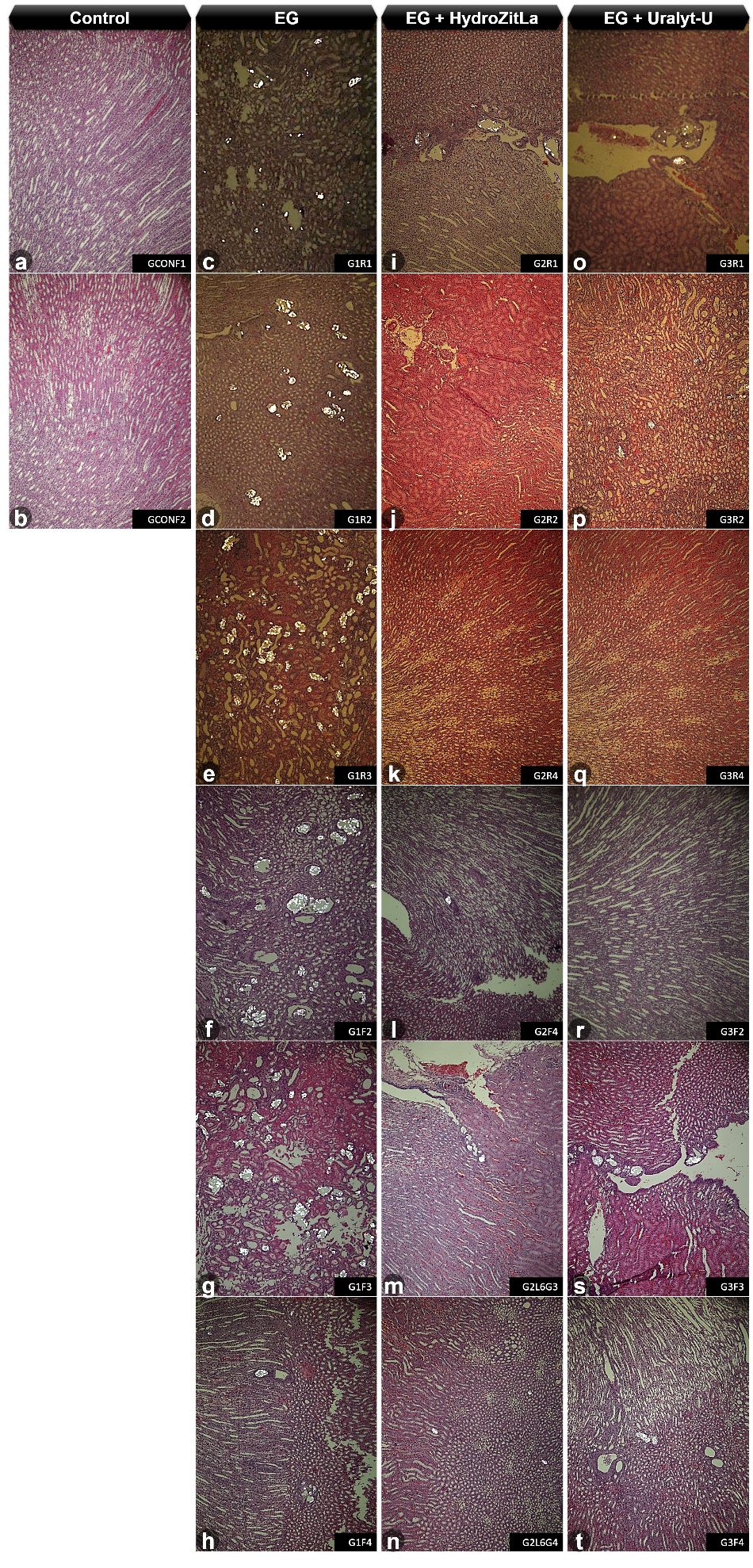


**Supplementary Figure 4** Yasue staining micrographs of all four groups of rat renal sections. Magnification: x100.


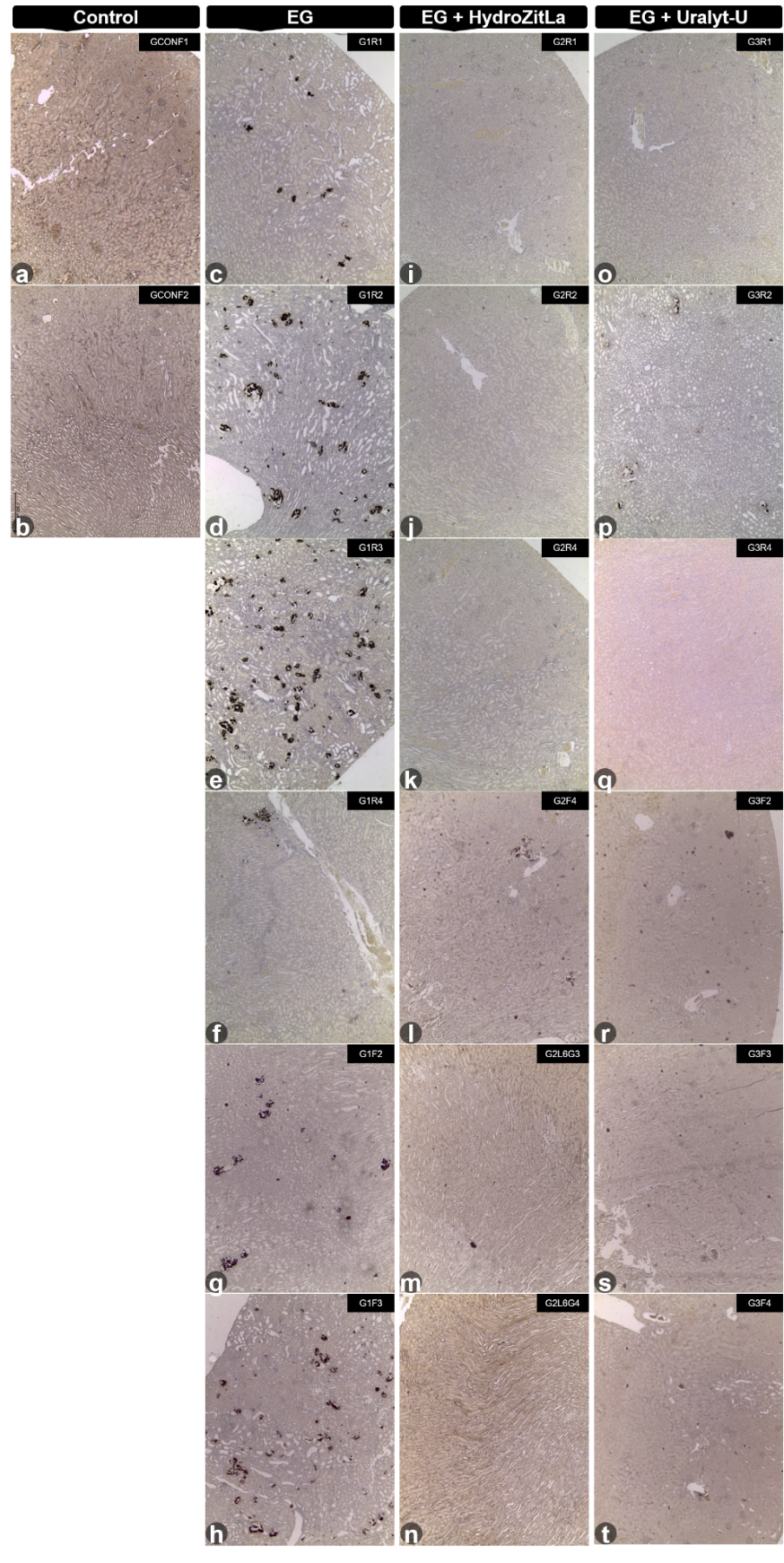


**Supplementary Figure 5** Representative micrographs of 4-HNE expression in the renal sections of all four groups of rats. Magnification: x400.


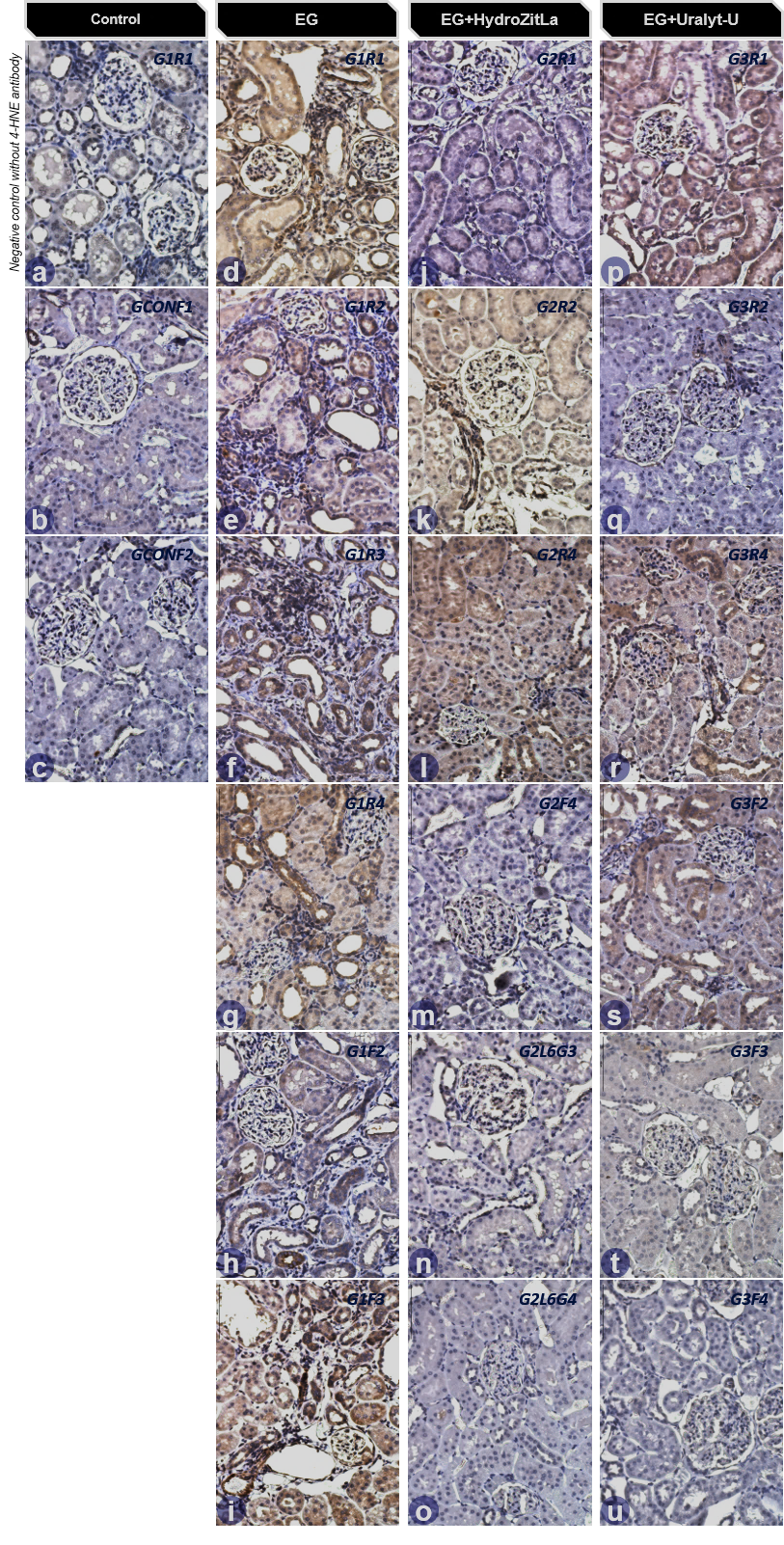


**Supplementary Figure 6** Representative micrographs of cleaved Caspase-3 expression in renal sections. Intrarenal expression of cleaved Caspase-3 in EG, EG + HydroZitLa, EG + Uralyt-U, and control rats were relatively comparable. Magnification: x400.


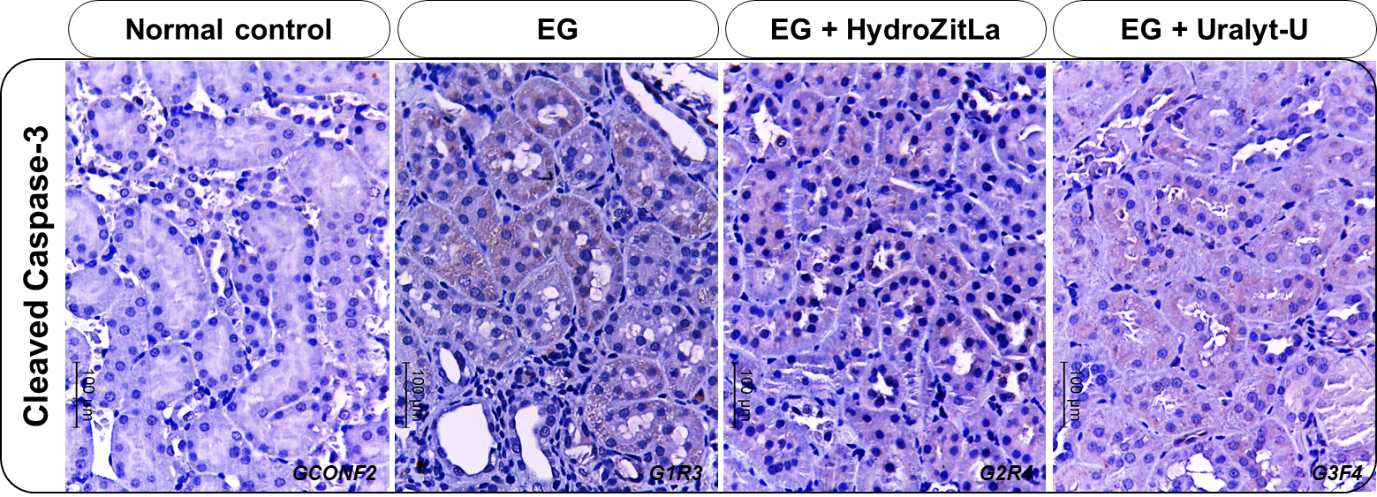


**Supplementary Figure 7** The effect of HydroZitLa (varied concentrations from 1 to 100% v/v) on *C. elegans* survival. At the extremely high doses (90 and 100% v/v), HydroZitLa (HZL) was evidently toxic to the *C. elegans*. HydroZitLa supplementation at 30% v/v delivered the most lifespan extending effect in tested nematodes.


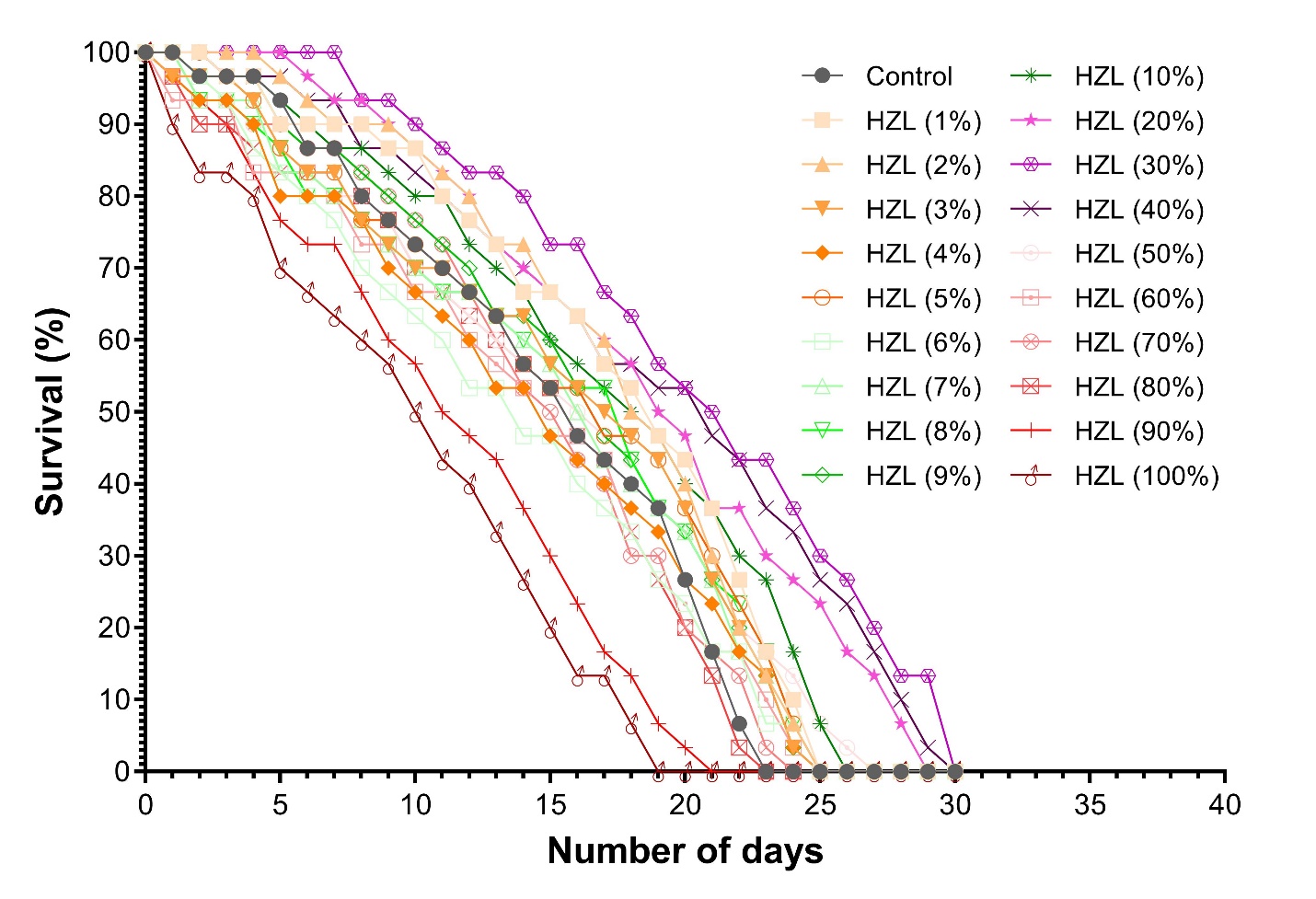


**Supplementary Figure 8** A: Total antioxidant capacity (TAC, measured by ABTS method) of HydroZitLa was 1.87 ± 7.96 mg vitamin C equivalent antioxidant capacity/pouch, but TAC in Uralyt-U drug was undetectable (UD). B: Total phenolic content (TPC) and total flavonoid content (TFC) of HydroZitLa were 19.99 ± 0.83 mg gallic acid equivalent/100 g, and 4.59 ± 0.31 mg catechin equivalent/100 g, respectively.


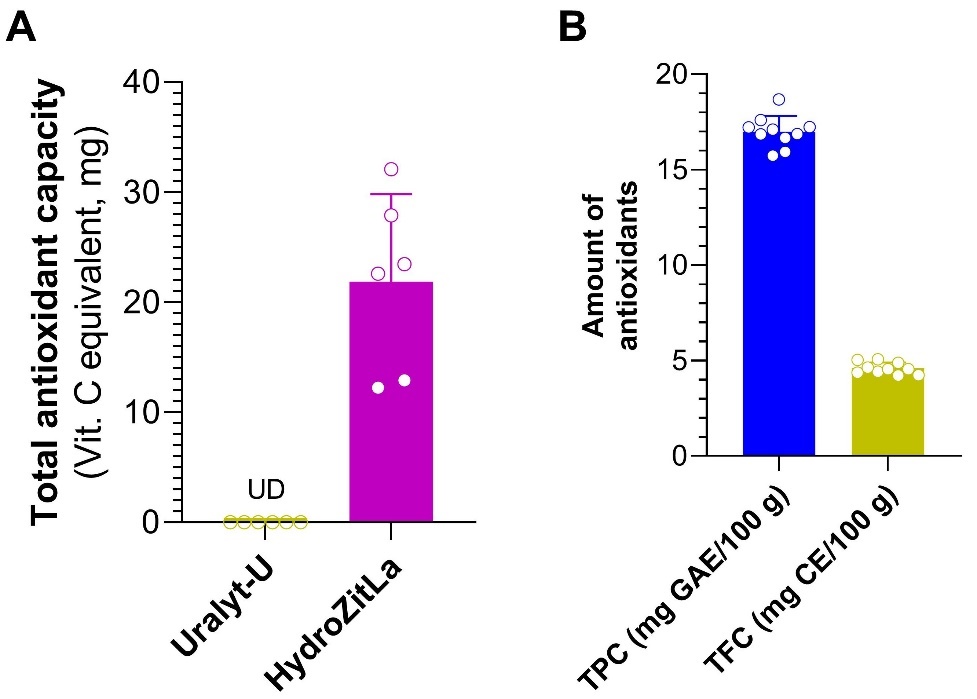

Supplement: Supplementary file 1 — Supplementary Information. [file 41598_2022_8316_MOESM1_ESM.doc]
